# Supplementary material for: Evaluation of the efficacy of Lactobacillus-containing feminine hygiene products on vaginal microbiome and genitourinary symptoms in pre- and postmenopausal women: A pilot randomized controlled trial
Source: PLoS One. 2022 Dec 30;17(12):e0270242. doi: 10.1371/journal.pone.0270242 (PMC9803311; doi:10.1371/journal.pone.0270242)
Supplement: S1 File — (PDF) [file pone.0270242.s001.pdf]

Simplified Menopausal Index Questionnaire

| Symptom | Severe | Moderate | Mild | Never | Scores |
|---|---|---|---|---|---|
| 1. Hot flashes | 10 | 6 | 3 | 0 | |
| 2. Sweats | 10 | 6 | 3 | 0 | |
| 3. Cold constitution | 14 | 9 | 5 | 0 | |
| 4. Shortness of breath or palpitation | 12 | 8 | 4 | 0 | |
| 5. Insomnia | 14 | 9 | 5 | 0 | |
| 6. Easy excitability or irritability | 12 | 8 | 4 | 0 | |
| 7. Worry about self or depression | 7 | 5 | 3 | 0 | |
| 8. Headache, vertigo or nausea | 7 | 5 | 3 | 0 | |
| 9. Easy fatigability | 7 | 4 | 2 | 0 | |
| 10. Shoulder stiffness, lumbago or joint pain | 7 | 5 | 3 | 0 | |
| Total scores | | | | | |

Interpretation of scores

0-25: You have been taking good care of your wellbeing. Please continue your current lifestyle habits.

26-50: Please take care of your diets, physical activities, and lifestyle habits.

51-65: We recommend consultation with the physician for counseling and treatment.

66-80: We recommend a treatment plan for more than 6 months.

81-100: We recommend thorough investigation of the underlying problems. If the problems are due to menopause, consultation with a gynecologist is required for a long-term treatment plan.
