# Supplementary material for: Evaluation of the efficacy of Lactobacillus-containing feminine hygiene products on vaginal microbiome and genitourinary symptoms in pre- and postmenopausal women: A pilot randomized controlled trial
Source: PLoS One. 2022 Dec 30;17(12):e0270242. doi: 10.1371/journal.pone.0270242 (PMC9803311; doi:10.1371/journal.pone.0270242)
Supplement: S2 File — (PDF) [file pone.0270242.s002.pdf]

Overactive bladder symptom scores (OABSS)

How often do you experience the following symptoms? Please circle the score that best applied to your urinary condition during the past week.

Item	Score	Frequency
Q1. How often do you typically urinate, from waking in the morning until sleeping at night?	0<br>1<br>2	≤7 times<br>8–14 times<br>≥15 times
Q2. How often do you typically wake up to urinate, from sleeping at night until waking in the morning?	0<br>1<br>2<br>3	None<br>Once<br>2 times<br>≥3 times
Q3. How often do you have a sudden desire to urinate, which is difficult to defer?	0<br>1<br>2<br>3<br>4<br>5	None<br>Less than once a week<br>Once a week or more<br>About once a day<br>2–4 times a day<br>≥5 times a day
Q4. How often do you leak urine because you cannot defer the sudden desire to urinate?	0<br>1<br>2<br>3<br>4<br>5	None<br>Less than once a week<br>Once a week or more<br>About once a day<br>2–4 times a day<br>≥5 times a day
Overall scores		

OAB was defined as urinary urgency once a week or more ($Q2 \geq 2$) and total score of OABSS ≥ 3.

Interpretation of overall scores
≥5, mild
6–11, moderate
≥12, severe

This is just a scoring symptom so if you have any urinary symptoms please consult with the physician.
