# Supplementary material for: Evaluation of the efficacy of Lactobacillus-containing feminine hygiene products on vaginal microbiome and genitourinary symptoms in pre- and postmenopausal women: A pilot randomized controlled trial
Source: PLoS One. 2022 Dec 30;17(12):e0270242. doi: 10.1371/journal.pone.0270242 (PMC9803311; doi:10.1371/journal.pone.0270242)
Supplement: S3 File — (PDF) [file pone.0270242.s003.pdf]

「乳酸菌配合膣内潤滑剤」を使用した
デリケートゾーンケアに関するモニター調査
（計画書）

調査実施責任者
吉形玲美　医師
医療法人社団進興会 浜松町ハマサイトクリニック
住所：東京都港区海岸 1-2-20　汐留ビルディング 2F
電話番号：03-5472-1100　FAX 番号：03-5472-3355

調査実施事務局
株式会社アドバンスト・メディカル・ケア　エイジングケア事業部
東京都港区六本木 7-15-14　塩業ビル 7 F
電話番号：03-6890-0036　FAX 番号：03-5413-1023
担当：椎名紋子 a-shiina@amcare.co.jp
　　　高野真未 m-takano@amcare.co.jp
　　　磯野昭次 s-isono@amcare.co.jp

作成：　2021 年 3 月 23 日　（最終版）

秘密保護について

モニター調査計画書は、本調査に関わる、倫理審査委員会、調査実施責任者、調査協力者、及び調査実施施設である浜松町ハマサイトクリニック、その付属各部署に限定して提供される秘密情報として取り扱うこととする。

1. 調査実施の背景・目的

女性ホルモンの減少やバランスの乱れが原因でデリケートゾーンの不快症状（かゆみ、におい、痛み等）を有する女性は決して少なくない。2017年に国内で実施された40歳以上の女性10,000人を対象としたWEB調査の結果では、45％の女性が何らかの泌尿器系の症状を有し、その中の79％が症状について心配しているとの結果であった。[1)] 近年、デリケートゾーンのケア商品がいくつか発売され、セルフメディケーションが普及しつつあるが、その有用性についてのエビデンスは十分ではない。今回、アドバンスト・メディカル・ケア（以下ＡＭＣ）が新たに商品開発したデリケートゾーンケア商品「乳酸菌配合腟内潤滑剤」を販売するに当たり、モニター調査を実施し、商品としての有用性を確認することはＡＭＣにとって重要な課題である。

2. モニター調査の方法

1） **調査方法：**従来のデリケートソープとクリーム（保湿剤）に加えて「乳酸菌配合腟内潤滑剤」を使用するデリケートゾーンケアが、デリケートソープとクリームのみのケアと比較して有用性に違いがみられるかを調査する。調査項目については、4週後に匿名化で入力したデータベースを基に評価する。中止・脱落例は評価対象外とする。

2） **目標症例数：70**例（未閉経の健常女性35例、閉経後の健常女性35例）。

①未閉経（35名）	**5名**	ソープ・クリーム・腟内潤滑剤のいずれも不使用
	**15名**	ソープとクリームでケア
	**15名**	ソープ、クリーム、腟内潤滑剤でケア
②閉経後（35名）	**5名**	ソープ・クリーム・腟内潤滑剤のいずれも不使用
	**15名**	ソープとクリームでケア
	**15名**	ソープ、クリーム、腟内潤滑剤でケア

3） **調査期間：**4週間（2021年5月～7月の期間中の4週間）

3. モニター調査で使用する商品と使用方法：

1） デリケートゾーンケアソープ（商品名：デリケートソフトウォッシュ/販売会社：ＡＭＣ）
入浴時に1回当たり2プッシュ使用しデリケートゾーンを洗浄する
主要成分：乳酸菌、ダイズ種子エキス、ダマスクバラ花エキス

2） デリケートゾーンケアクリーム（商品名：デリケートソフトジェルクリーム/販売会社：ＡＭＣ）。入浴後に2㎝径を手に取り大陰唇を中心に塗布する
主要成分：乳酸菌、ダイズ種子エキス、ソメイヨシノ葉エキス

3） 乳酸菌配合腟内潤滑剤（商品名：未定/販売会社：ＡＭＣ予定）
3日に1回（推奨使用回数）就寝前に腟内に挿入する
主要成分：乳酸菌、ヒアルロン酸Na、乳酸、クエン酸Na
株式会社ハナミスイが2014年1月から販売しているインクリア®（既に国内で1700万個以上の販売実績）をベースとし、新たに乳酸菌を配合した腟内潤滑剤。ハナミスイとの共同で開発。

4. 商品の安全性

現時点で、デリケートソフトウォッシュ、デリケートソフトジェルクリームについて有害事象は報告されていない。また、乳酸菌配合腟内潤滑剤のベースであるインクリア®は、株式会社ハナミスイが生体適合性試験で安全性を確認した後 2014 年 1 月に販売し、現在に至るまで有害事象は報告されていない。なお、新規に配合する乳酸菌については、原材料メーカーが動物実験、生体適合性試験で安全性を確認している。

5. 調査対象者

1） **対象者**：20 歳以上 49 歳以下の未閉経の健常女性 35 例、50 歳以上 75 歳以下の閉経後の健常女性 35 例の計 70 例で本調査の参加に同意が得られた者。閉経の定義は、自然閉経（月経が過去 1 年以上ないこと）かつ、FSH25mIU/mL 以上、E2（エストラジオール）20pg/mL 未満とし手術等の侵襲による閉経は除く。

2） **除外基準：**

① 尿路感染症で治療中の患者
② 尿路結石、水腎症、尿路腫瘍などにより泌尿器科的処置を受けている患者
③ 調査開始前（2 週間)、調査期間中に抗生物質およびステロイド剤を服用した者
④ 外陰皮膚粘膜に明らかな皮膚科（婦人科）疾患及び腟炎等の婦人科疾患を有する者（無症状の萎縮性腟炎は除く）
⑤ その他、調査実施医師が適切でないと認めた者

3） **登録：**調査実施医師は、候補となる参加者が適格基準を満たし、除外基準のいずれにも該当しないことを確認した上で、文書同意を得た後に参加者として登録する。

4） **中止・脱落：下記に該当する場合は、モニター調査からの脱落症例とする。**

① 参加者の希望により本調査の参加を辞退したいと申し入れがあった時
② 参加者が調査計画書で決められたデリケートゾーンケアの実施率が 50%以下の場合
③ 抗生物質およびステロイド剤を調査期間中に服用した場合
④ 調査期間中、前述の 5 調査対象者の 2）除外基準の項に定める事象が発生した時

5） **調査の中止基準：下記に該当する場合は、モニター調査を中止する。**

① 調査期間中、参加者に重篤な症状が発症し、被験製品の安全性に問題が生じた場合
② 被験製品の継続使用により、参加者が何らかの不利益を被ることが明らかになった場合

6. 調査項目

検査項目	0週	1週	2週	3週	4週
① 膣内細菌叢検査	●				●
② 膣内pH検査	●				●
③ アンケート調査	●				●
・不快症状の有無・種類	●				●
・生活習慣	●				
・商品の使用状況・使用感					●
④ 膣細胞成熟度指数	○				○
⑤ 卵巣機能検査（E2、FSH）	○				
⑥ 腸内細菌叢検査	○				
⑦ エクオール産生能検査	○				

●：主要評価項目

① 膣内細菌叢検査：調査担当医師が、膣内のぬぐい液を専用キットで採取する。外部検査会社が次世代シーケンサーを用いて膣内細菌の種類の同定と割合（ラクトバチルス属種類の保有率・占有率）及び多様性指数、クラスター分析を解析する。

② 膣内 pH 検査：調査担当医師が専用の pH キットを用いて膣内 pH を測定する。

③ アンケート調査：調査開始前と終了後に参加者が指定のアンケート用紙に記入する。

○：副次的評価項目

④ 膣細胞成熟度指数：調査担当医師が内膜細胞診と同様にブラシで粘膜を採取する。外部検査会社が傍基底細胞・中層細胞・表層細胞の各細胞から比率を算出する。

⑤ 卵巣機能検査：採血にて E2、FSH を測定する。

⑥ 腸内細菌検査：専用のキットを使用して採便し、外部検査会社が菌種の同定と割合を解析する。

⑦ エクオール産生能検査：参加者の検尿から外部検査会社がエクオール量を測定する。

7. 評価方法

主要評価項目については群間比較、前後比較にて x2 検定、T 検定を行う。アンケート調査結果については、症状については前後比較、その他のアンケート調査結果については主要評価項目との相関関係を評価する。副次的評価項目については、匿名コード化した検査値をデータベースに入力し相関関係を分析する。中止・脱落症例、欠測値については、解析対象に含まない。

8. 参加者の同意取得

本モニター調査の開始に先立ち、調査担当医師は参加希望者に対して、調査の目的、内容、その他必要事項を説明文書に基づいて説明した後、参加希望者の自由意思に基づいた同意を文書により得る。参加者は、同意書を提出した後、また調査開始後であってもいつでも辞退できるものとする。

9. 有害事象の取扱い

調査中に発現した好ましくない或いは意図しない徴候、症状が認められた場合は、本調査との因果関係の有無に係わらず、原則として正常化または有害事象として捉えないレベルに回復する

まで追跡調査を行う。ただし、調査実施責任医師が回復と判断した場合はその限りではない。

10. 参加者への補償

本調査に起因して、参加者の身体に何らかの症状や不調が現れた場合は、調査担当医師が速やかに適切な診察や治療等、最善と思われる措置を行う。本調査との関わりが明らかな場合は、参加者の治療等に要する自己負担はなしとする。なお、健康被害の補償或いは賠償責任が生じた場合は、調査実施依頼会社であるアドバンスト・メディカル・ケアが補償します。

11. 参加者への負担軽減費の支払い

参加者には、別紙（参加者募集のご案内）に定める受診のための交通費及び負担軽減費を調査終了後に支払うこととする。

12. 個人情報の保護

個人情報保護にあたっては、本調査に関わるデータを全て匿名化（コード化）し、取り扱うことを基本とする。なお、特定の個人が識別可能な情報については、個人情報管理責任者の厳密な管理のもと、アドバンスト・メディカル・ケア内の施錠できる場所にて保管管理する。不要になった際には焼却・溶解等、再現不能な形で処分する。

13. モニター調査実施施設

医療法人社団進興会 浜松町ハマサイトクリニック

14. 調査実施責任医師

吉形玲美　医師
医療法人社団進興会 浜松町ハマサイトクリニック
住所：東京都港区海岸 1-2-20　汐留ビルディング 2F
電話番号：03-5472-1100　FAX 番号：03-5472-3355

15. 個人情報管理責任者

國枝真理
医療法人社団進興会 浜松町ハマサイトクリニック
住所：東京都港区海岸 1-2-20　汐留ビルディング 2F
電話番号：03-5472-1100　FAX 番号：03-5472-3355

16. 調査実施事務局・連絡先

株式会社アドバンスト・メディカル・ケア　エイジングケア事業部
東京都港区六本木 7-15-14　塩業ビル７F
電話番号：03-6890-0036　FAX 番号：03-5413-1023
担当：椎名紋子、高野真未、磯野昭次

17. 予算

No.	費用内訳	単価	例数	回数・個数	費用
1	膣内細菌検査	¥10,000	70	2	¥1,400,000
2	膣pH測定	¥1,500	70	2	¥210,000
3	膣細胞成熟度指数	¥1,500	70	2	¥210,000
4	卵巣機能検査（E2、FSH）	¥4,400	70	1	¥308,000
5	腸内細菌検査	¥10,000	70	1	¥700,000
6	エクオール産生能検査	¥3,000	70	1	¥210,000
7	調査使用製品（ソフトウォッシュ）	¥552	60	1	¥33,120
8	調査使用製品（ジェルクリーム）※	¥752	60	1	¥45,120
9	調査使用製品（インクリア新処方）	¥730	30	1	¥21,900
10	参加者負担軽減費 ①(未閉経)	¥5,000	35	1	¥175,000
11	参加者負担軽減費 ②(閉経後)	¥10,000	35	1	¥350,000
12	交通費	¥1,000	70	2	¥140,000
13	モニター調査施設協力費	¥10,000	70	1	¥700,000
14	解析費用	¥50,000		1	¥50,000
	合計 ※原価				¥4,553,140

引用論文：

1）H. Ohta et al. Online survey of genital and urinary symptoms among Japanese women aged between 40 and 90 years. Climacteric, DOI: 10.1080/13697137.2020. https://doi.org/10.1080/13697137.2020.1768236

2）J.Revel et al. Vagina microbiome of reproductive-age women, PNAS 2011.

3）Ana Elisa Ribeiro et al. Can the use of probiotics in association with isofllavone improve the symptoms of genitourinary syndrome of menopause? Results from a randomized controlled trial. Menopause, Vol. 26, No.6, 2019
