# Supplementary material for: Evaluation of the efficacy of Lactobacillus-containing feminine hygiene products on vaginal microbiome and genitourinary symptoms in pre- and postmenopausal women: A pilot randomized controlled trial
Source: PLoS One. 2022 Dec 30;17(12):e0270242. doi: 10.1371/journal.pone.0270242 (PMC9803311; doi:10.1371/journal.pone.0270242)
Supplement: S4 File — (PDF) [file pone.0270242.s004.pdf]

Using "*Lactobacillus* containing Vaginal Lubricant"

Survey on feminine care

(Research Proposal)

Lead Researcher Physician
Dr. Remi Yoshikata
Hamamatsucho Hamasite Clinic, Shinkokai Medical Corporation
Address: Shiodome Building 2F, 1-2-20 Kaigan, Minato-ku, Tokyo
Phone number: 03-5472-1100 Fax number: 03-5472-3355

Implementation Office
Advanced Medical Care Co., Ltd. Aging Care Division
7-15-14 Engyo Building 7F, Roppongi, Minato-ku, Tokyo
Phone number: 03-6890-0036 FAX number: 03-5413-1023
Persons in charge:
Ayako Shiina a-shiina@amcare.co.jp
Mami Takano m-takano@amcare.co.jp
Akiji Isono s-isono@amcare.co.jp

Created: March 23, 2021 (final version)

1. Background and objective

Women sometimes have unpleasant symptoms (itchiness, bad odor, pain, etc.) in their private areas due to the decrease or imbalance of female hormones. According to a 2017 online survey of 10,000 women aged 40 and over in Japan, 45% of women had some urinary symptoms, and 79% of them were worried about their symptoms.[1)] In recent years, several feminine care products have been released and self-medication is becoming popular, but there is not enough evidence for its usefulness. In this study, we aimed to confirm the usefulness of a feminine care product " Lactobacillus containing Vaginal Lubricant", newly developed by Advanced Medical Care (hereinafter referred to as AMC).

2. Methods

① Survey method: Feminine care using "equol lactobacillus-containing vaginal lubricant" in addition to conventional delicate soap and cream (moisturizer) is different in usefulness compared to care with only delicate soap and cream. Investigate whether you can see The survey items will be evaluated based on the anonymized database input after 4 weeks. Discontinued/dropped cases will not be evaluated.

② Target sample size: 70 cases (35 non-menopausal healthy women, 35 post-menopausal healthy women).

1. Premenopausal women	5	Control
	15	Soap and cream
	15	Soap, cream, and lubricant
2. Postmenopasual women	5	Control
	15	Soap and cream
	15	Soap, cream, and lubricant

③ Survey period：4 weeks (May to July 2021)

3. Products used in the monitor survey and how to use them:

1) Feminine care Soap (Product name: Delicate Soft Wash/Distributor: AMC)
Use 2 pushes per time when bathing to wash the private area
Main ingredients: *Lactobacillus*, soybean seed extract, damask rose flower extract

2) Feminine care Cream (Product name: Delicate Soft Gel Cream/Distributor: AMC). After bathing, pick up a 2cm diameter amount and apply it to the labia majora.
Main ingredients: *Lactobacillus*, soybean seed extract, Yoshino cherry leaf extract

3) Vaginal lubricant containing lactic acid bacteria (Product name: undecided / Sales company: AMC planned)
Insert into the vagina once every 3 days (recommended number of uses) before going to bed
Main ingredients: *Lactobacillus*, sodium hyaluronate, lactic acid, sodium citrate
Based on Inclear®, which has been sold by Hanamisui Co., Ltd. since January 2014 (already sold more than 17 million units in Japan), it is a new vaginal lubricant containing *Lactobacillus*.

Developed jointly with Hanamisui.

4. Product safety

To date, no adverse events have been reported for Delicate Soft Wash and Delicate Soft Gel Cream. Hanamisui Co., Ltd. launched Inclear®, the base of the vaginal lubricant containing *Lactobacillus*, in January 2014 after confirming its safety in biocompatibility tests, and no adverse events have been reported to date. The manufacturer has confirmed the safety of the newly added *Lactobacillus* through animal experiments and biocompatibility tests.

5. Survey Subjects

1) Subjects: 35 non-menopausal healthy women aged 20 to 49 and 35 healthy postmenopausal women aged 50 to 75, a total of 70 who consented to participate in this survey. Menopause is defined as spontaneous menopause (absence of menstruation for the past 1 year or more), FSH 25mIU/mL or more, and E2 (estradiol) less than 20pg/mL, excluding menopause due to invasive surgery.

2) Exclusion Criteria:

① Patients undergoing treatment for urinary tract infections

② Patients undergoing urological treatment due to urinary tract stones, hydronephrosis, urinary tract tumors, etc.

③ Those who took antibiotics and steroids before the survey (2 weeks) and during the survey period

④ Subjects with obvious dermatological (gynecological) diseases in the vulvar skin mucosa and gynecological diseases such as vaginitis (excluding asymptomatic atrophic vaginitis)

⑤ In addition, those who are deemed inappropriate by the investigator

3) Enrollment: The investigator will confirm that the candidate meets the eligibility criteria and does not meet any of the exclusion criteria, and will enroll them as participants after obtaining written informed consent.

4) Withdrawal: Patients who fall under any of the following will be withdrawn from the monitoring survey.

① When there is a request to decline participation in this survey at the request of the participant

② If the participant's implementation rate of feminine care determined in the research plan is 50% or less

③ Taking antibiotics and steroids during the investigation period

④ During the investigation period, when any of the 5 conditions specified in 2) Exclusion Criteria occurs.

5) Survey discontinuation criteria: Monitor surveys will be discontinued if any of the following apply.

(1) If a participant develops a serious symptom during the study period and the safety of the test

product is questioned.

(2) When it becomes clear that the participant suffers some kind of disadvantage due to the continued use of the test product.

6. Outcome measures

Measures	Baseline	Week 1	Week 2	Week 3	Week 4
**1.** Vaginal microflora test	●				●
**2.** Vaginal pH	●				●
**3.** Questionnaires	●				●
Symptom assessment	●				●
Lifestyle	●				
Product review					●
**4.** Vaginal maturation index	○				○
**5.** Estradiol, Follicular Stimulating Hormone serum titers	○				
**6.** Gut microflora test	○				
**7.** Equol producing ability test	○				

● : Primary endpoint

(1) Vaginal flora test: The investigator will collect a vaginal swab using a special kit. An external testing company will use a next-generation sequencer to identify and rate the types of vaginal bacteria (prevalence/occupancy rate of Lactobacillus species), diversity index, and cluster analysis.

(2) Vaginal pH test: The investigator will measure the vaginal pH using a dedicated pH kit.

(3) Questionnaire survey: Participants fill out a designated questionnaire before and after the survey.

○: Secondary endpoint

④ Vaginal cell maturity index: The investigator collects the mucous membrane with a brush in the same way as endometrial cytology. An external testing company calculates the ratio from each cell of parabasal cells, middle layer cells, and superficial cells.

⑤ Ovarian function test: Measure E2 and FSH by blood sampling.

⑥ Intestinal bacteria test: Stool is collected using a dedicated kit, and an external testing company identifies the bacterial species and analyzes the ratio.

⑦ Equol production capacity test: An external testing company measures the amount of equol from the participants' urine.

7. Data analysis

For the primary endpoint, chi-squared test and t-test will be performed for inter-group comparison and before-and-after comparison. Regarding the questionnaire survey results, the symptoms will be compared before and after using the products. The correlation with the primary endpoint will be evaluated for other questionnaire survey results. For secondary endpoints, we will find out correlations among anonymously

coded lab test values. Drop-out cases and missing values will not be included in the analysis.

8. Obtaining consent

Prior to the start of this survey, the investigator explained the purpose, content, and other necessary matters of the survey to the applicants based on the explanatory document, and then obtain written consent based on the applicants' free will. Participants may withdraw at any time after submitting the consent form and even after the survey has begun.

9. Handling of Adverse Events

If we observe any unfavorable or unintended signs or symptoms that appear during the study, follow-up will be conducted until they recover to a level that cannot be treated as an adverse event, regardless of whether or not there is a causal relationship with this study. However, this does not apply if the lead researcher physician determines that the patient has recovered.

10. Compensation for Participants

In the event that a participant develops any symptoms or discomfort as a result of this survey, the doctor in charge of the survey will promptly take the best possible measures, such as appropriate examination and treatment. If the relationship with this survey is clear, the participant shall not bear any treatment cost. In the event that compensation for health damage or liability arises, Advanced Medical Care, the company funding the survey, will compensate.

11. Payment of burden reduction fee to participants

We will pay transportation and medical exam expenses as specified in the attached sheet.

12. Protection of personal information

All data related to this survey will be anonymized (coded) in order to protect personal information. Information that can identify a specific individual will be stored and managed in a locked area within Advanced Medical Care under the strict control of the person responsible for managing personal information. When it is no longer needed, it will be handled in an irreproducible manner such as incineration or dissolution.

13. Research site

Hamamatsucho Hamasite Clinic, Shinkokai Medical Corporation

14. Lead researcher physician

Dr. Remi Yoshigata
Hamamatsucho Hamasite Clinic, Shinkokai Medical Corporation
Address: Shiodome Building 2F, 1-2-20 Kaigan, Minato-ku, Tokyo
Phone number: 03-5472-1100 Fax number: 03-5472-3355

15. Privacy information manager

Mari Kunieda

Hamamatsucho Hamasite Clinic, Shinkokai Medical Corporation

Address: Shiodome Building 2F, 1-2-20 Kaigan, Minato-ku, Tokyo

Phone number: 03-5472-1100 Fax number: 03-5472-3355

16. Survey Implementation Office/Contact

Advanced Medical Care Co., Ltd. Aging Care Division

7-15-14 Engyo Building 7F, Roppongi, Minato-ku, Tokyo

Phone number: 03-6890-0036 FAX number: 03-5413-1023

Persons in charge: Ayako Shiina, Mami Takano, Shoji Isono

17. Budget

No.	Item	Unit cost (yen)	Units	Amount	Total
1.	Vaginal microflora test	10000	70	2	1400000
2.	Vaginal pH	1500	70	2	210000
3.	Vaginal maturation index	1500	70	2	210000
4.	Estradiol, Follicular Stimulating Hormone serum titers	4400	70	1	308000
5.	Gut microflora test	10000	70	1	700000
6.	Equol producing ability test	3000	70	1	210000
7.	Softwash	552	60	1	33120
8.	Cream	752	60	1	45120
9.	Lubricant	730	30	1	21900
10.	Incentive 1(premenopausal women)	5000	35	1	175000
11.	Incentive 1(postmenopausal women)	10000	35	1	350000
12.	Transportation	1000	70	2	140000
13.	Collaboration fee	10000	70	1	700000
14.	Analysis	50000		1	50000
	Total				

References

１）H. Ohta et al. Online survey of genital and urinary symptoms among Japanese women aged between 40 and 90 years. Climacteric, DOI: 10.1080/13697137.2020. https://doi.org/10.1080/13697137.2020.1768236

２）J.Revel et al. Vagina microbiome of reproductive-age women, PNAS 2011.

３）Ana Elisa Ribeiro et al. Can the use of probiotics in association with isofllavone improve the symptoms of genitourinary syndrome of menopause? Results from a randomized controlled trial. Menopause, Vol. 26, No.6，2019
