# Supplementary figures and images for: Evaluation of the efficacy of Lactobacillus-containing feminine hygiene products on vaginal microbiome and genitourinary symptoms in pre- and postmenopausal women: A pilot randomized controlled trial
Source: PLoS One. 2022 Dec 30;17(12):e0270242. doi: 10.1371/journal.pone.0270242 (PMC9803311; doi:10.1371/journal.pone.0270242)

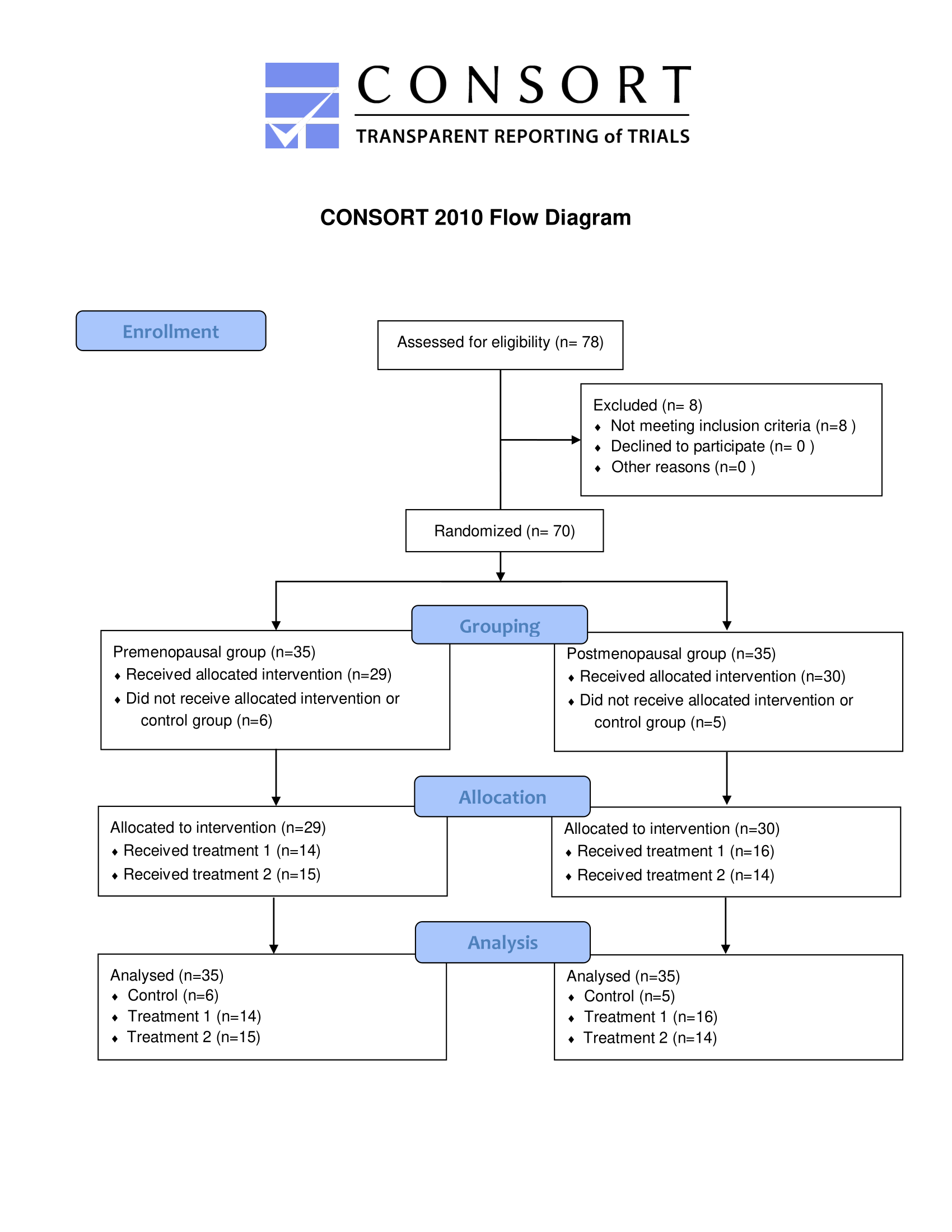

Supplement: S6 File — (ZIP) [file pone.0270242.s006.zip › Figures/Fig1.tiff]

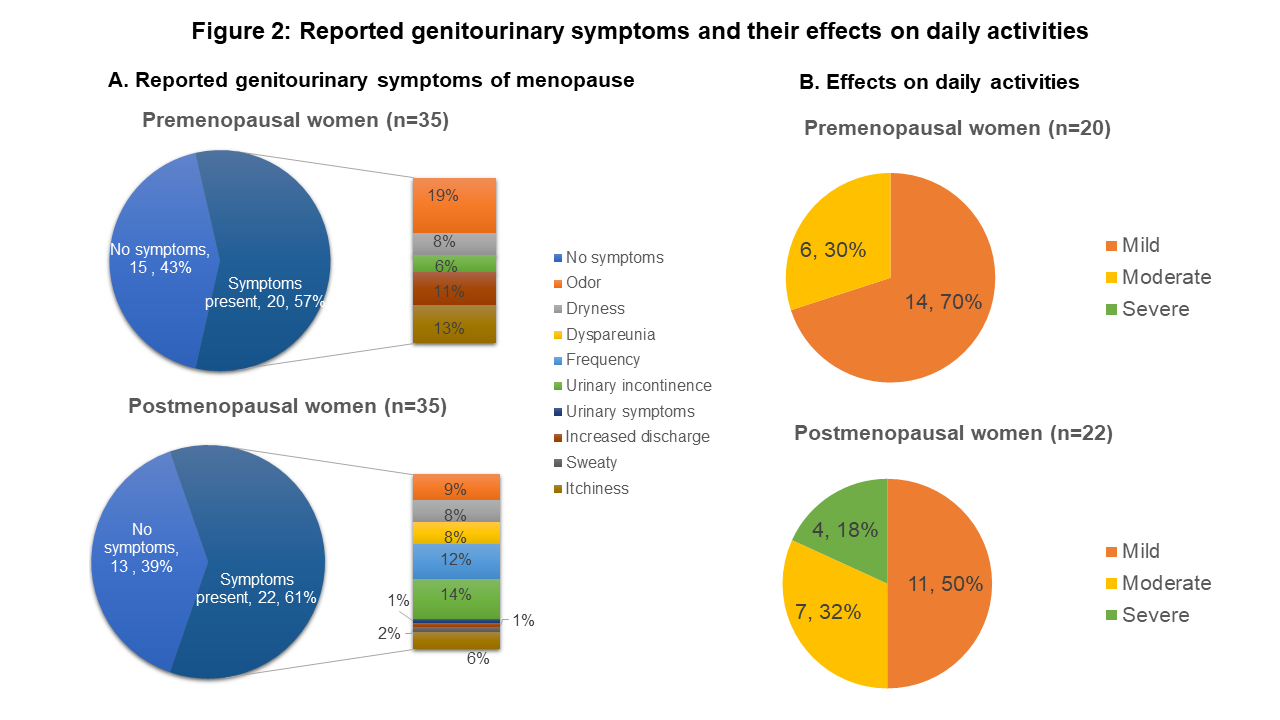

Supplement: S6 File — (ZIP) [file pone.0270242.s006.zip › Figures/Fig2.tif]

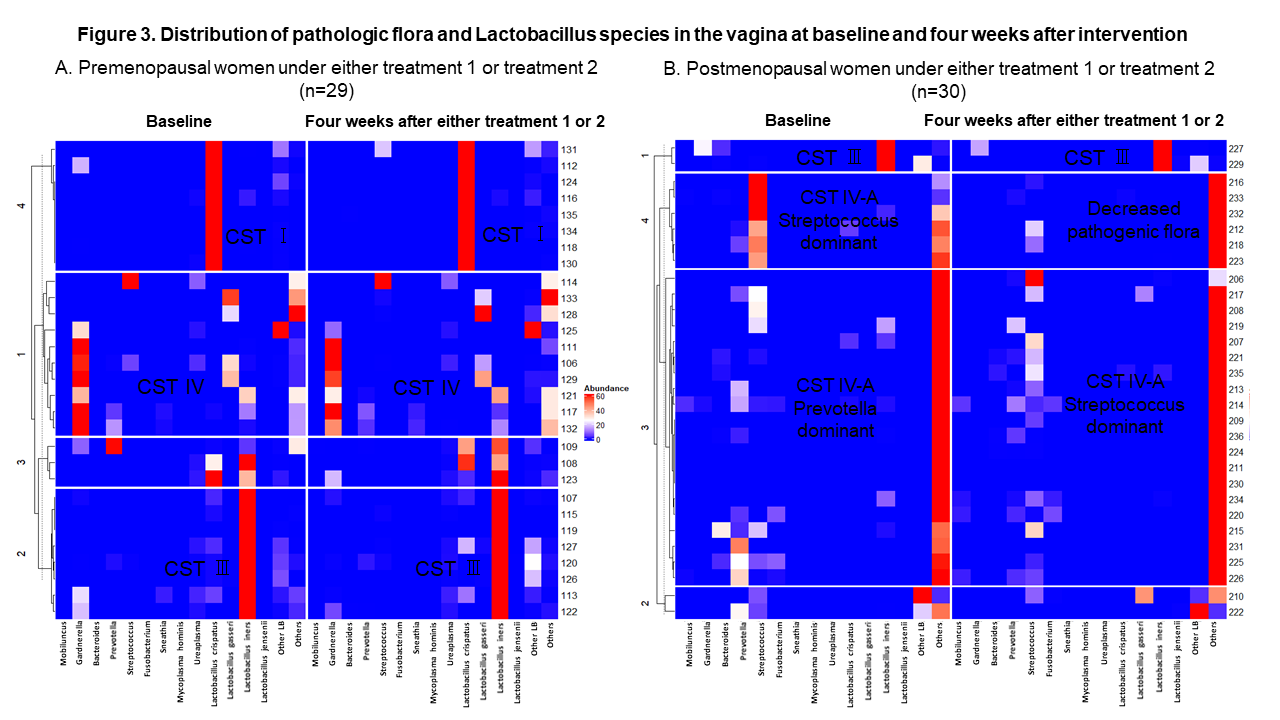

Supplement: S6 File — (ZIP) [file pone.0270242.s006.zip › Figures/Fig3.tif]

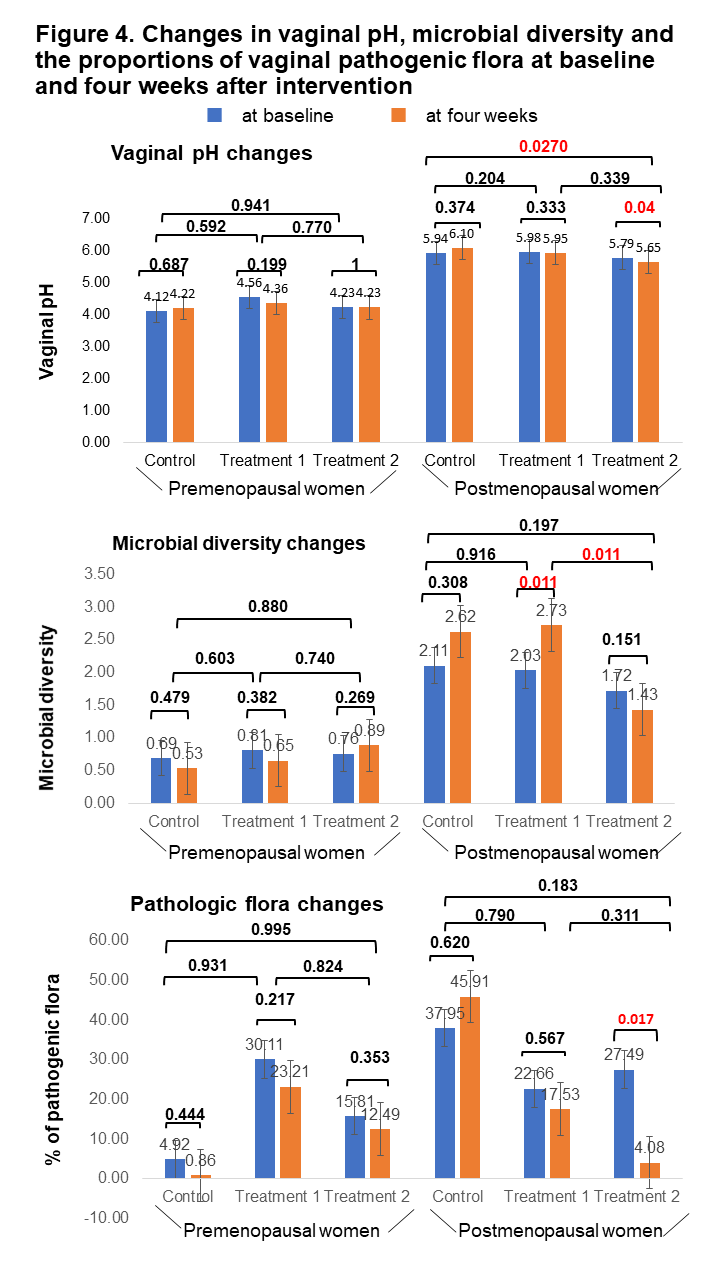

Supplement: S6 File — (ZIP) [file pone.0270242.s006.zip › Figures/Fig4.tif]

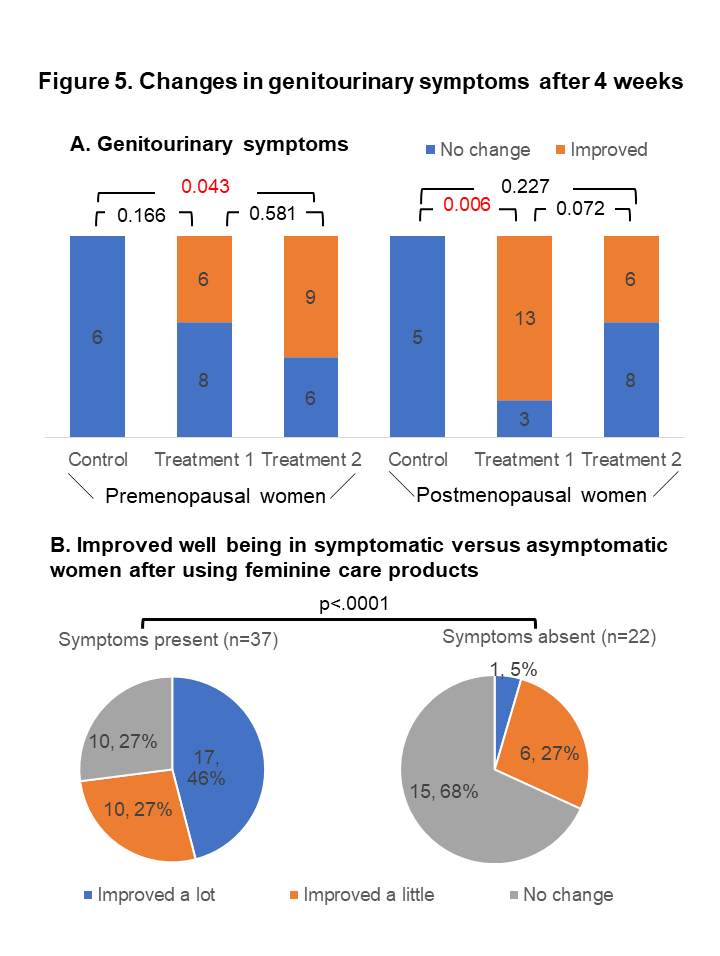

Supplement: S6 File — (ZIP) [file pone.0270242.s006.zip › Figures/Fig5.tif]

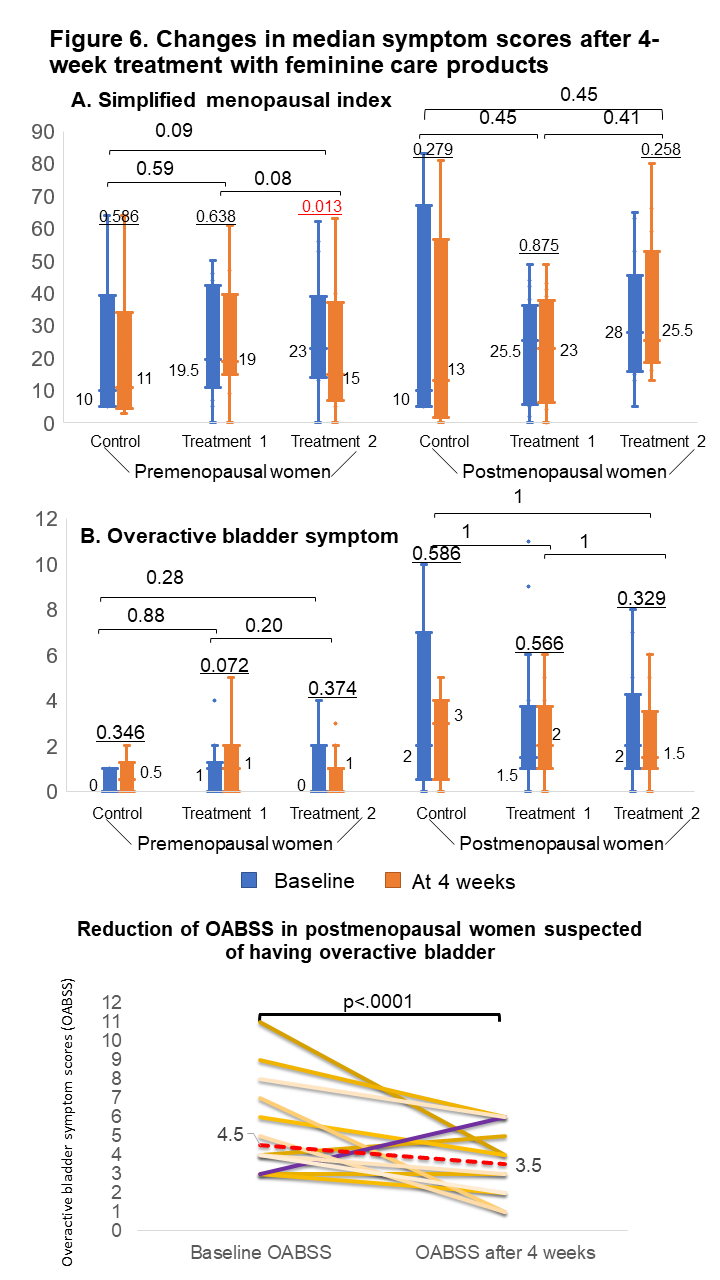

Supplement: S6 File — (ZIP) [file pone.0270242.s006.zip › Figures/Fig6.tif]
